# Hair Growth Promoting Effect of 4HGF Encapsulated with PGA Nanoparticles (PGA-4HGF) by β-Catenin Activation and Its Related Cell Cycle Molecules

**DOI:** 10.3390/ijms20143447

**Published:** 2019-07-13

**Authors:** Hye-Ji Lee, Ha-Kyoung Kwon, Hye Su Kim, Moon Il Kim, Hye-Jin Park

**Affiliations:** 1 Department of Food Science and Biotechnology, College of BioNano Technology, Gachon University, Gyeonggi-do 13120, Korea; 2 Department of BioNano Technology, College of BioNano Technology, Gachon University, Gyeonggi-do 13120, Korea

**Keywords:** PGA-4HGF nanoparticles, hair growth, dermal papilla cells, C57BL/6N, anagen phase, G_1_/S phase progression, β-catenin, cyclinD1, CDK4

## Abstract

Poly-γ-glutamic acid (γ-PGA)-based nanoparticles draw remarkable attention as drug delivery agents due to their controlled release characteristics, low toxicity, and biocompatibility. 4HGF is an herbal mixture of *Phellinus linteus* grown on germinated brown rice, *Cordyceps militaris* grown on germinated soybeans, *Polygonum multiflorum*, *Ficus carica*, and *Cocos nucifera* oil. Here, we encapsulated 4HGF within PGA-based hydrogel nanoparticles, prepared by simple ionic gelation with chitosan, to facilitate its penetration into hair follicles (HFs). In this study, we report the hair promoting activity of 4HGF encapsulated with PGA nanoparticles (PGA-4HGF) and their mechanism, compared to 4HGF alone. The average size of spherical nanoparticles was ~400 nm in diameter. Continuous release of PGA-4HGF was observed in a simulated physiological condition. As expected, PGA-4HGF treatment increased hair length, induced earlier anagen initiation, and elongated the duration of the anagen phase in C57BL/6N mice, compared with free 4HGF treatment. PGA-4HGF significantly increased dermal papilla cell proliferation and induced cell cycle progression. PGA-4HGF also significantly increased the total amount of β-catenin protein expression, a stimulator of the anagen phase, through induction of cyclinD1 and CDK4 protein levels, compared to free 4HGF treatment. Our findings underscore the potential of PGA nanocapsules to efficiently deliver 4HGF into HFs, hence promoting hair-growth. Therefore, PGA-4HGF nanoparticles may be promising therapeutic agents for hair growth disorders.

## 1. Introduction

Alopecia, also known as hair loss, is a condition caused by various factors such as hormone imbalance, stress, malnutrition, and chemotherapy [1,2,3]. Although hair loss is non-lethal, it has a profound effect on social interactions and psychological stability [4]. FDA-approved minoxidil and finasteride are widely used for treating hair loss [5]. However, undesired side effects, such as hypotension, dizziness, and tachycardia loss, limit their usage [6]. In this regard, there have been many attempts to target hair loss by developing novel pharmacological therapeutic agents sourced from traditional herbal medicines, free of side effects [7]. In this study, we explored a mixture of herbal extracts (4HGF) including *Phellinus linteus* grown on germinated brown rice, *Cordyceps militaris* grown on germinated soybeans, *Polygonum multiflorum*, *Ficus carica*, and *Cocos nucifera* oil. Several groups have reported the beneficial effects of *P. multiflorum* on hair-growth [7,8], and *C. nucifera* oil on skin/hair [9,10]. *P. linteus* grown on germinated brown rice [11], *C. militaris* grown on germinated soybeans [12,13], and *F. carica* [14] equally possess anti-inflammatory properties relevant to promoting hair growth. However, patients with hair loss are resistant to these natural products, owing to low absorption and poor hair-growth induction by the hair products on the market [7].

Recently, natural biodegradable polymer-based nanoparticles have become the center of attention in pharmaceutical and cosmetic industries [15]. Poly-γ-glutamic acid (γ-PGA)/chitosan nanoparticles are extensively used in many commercial applications such as drug, cosmetics, and dermatological products [16,17,18]. γ-PGA, a biodegradable, hydrophilic, and nontoxic polymer, is used in the preparation of nanoparticles through ionic gelation with chitosan, which is equally biodegradable, nontoxic, and exhibits antibacterial properties [19,20,21]. γ-PGA also improves drug penetration across cells and tissues and prolongs drug duration in the blood stream [15,22,23]. To increase the adsorption and transmission of hair-growth agents into the scalp, we applied a drug delivery technology that encapsulates hair-growth effective drugs or extracts into nanoparticles. Among them, PGA/chitosan nanoparticles are capable of entrapping biomolecules or agents into internal structures or absorbing them on their external surfaces [22,24]. They can also alter biological activities due to their large surface to volume ratio [22,24]. In addition, topical application of γ-PGA can promote hair-growth by moving hair follicle (HF) states from the telogen to anagen phase [25]. Therefore, we investigated the hair-growth effect of 4HGF encapsulated in PGA/chitosan nanoparticles (PGA-4HGF) in vitro and in vivo.

## 2. Results

### 2.1. Preparation, Characterization and Release Amount of PGA-4HGF

**Figure 1:**
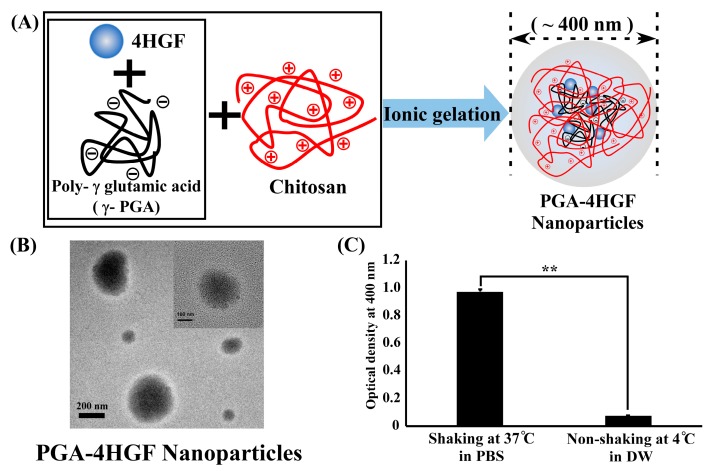
(**A**) Scheme of the complex formation between the polyanions (poly-γ-glutamic acid (γ-PGA)) and the polycation (chitosan) and the capsulation for 4HGF into PGA/chitosan nanoparticles. (**B**) TEM images of PGA-4HGF nanoparticles (left, PGA-4HGF, scale bar = 200 nm; right, PGA-control, scale bar = 100 nm). (**C**) Comparison of optical density from the release of 4HGF entrapped within PGA-4HGF after 6 h incubation at different conditions. Data was analyzed with paired *t*-test (** *p* < 0.01).

We synthesized the PGA-4HGF hydrogel nanoparticles simply via ionic gelation (Figure 1A). Spherical nanoparticles were clearly observed by TEM (Figure 1B), but their surface was irregular, presumably due to the complex salt-based ingredients of 4HGF. Although the size distributions were relatively broad, ranging up to 1000 nm, average hydrodynamic size was estimated to ~400 nm, which might be helpful to pass through the skin gap. We also evaluated the released amount of 4HGF from the nanoparticles by measuring the distinct absorbance corresponding to 4HGF. The results showed that the optical density (OD) value at 400 nm of 4HGF from PGA-4HGF was 0.97 ± 0.016 in a shaking water bath at 37 °C in phosphate-buffered saline (PBS), which simulated topical application by rubbing, while the OD value was 0.07 ± 0.002 without shaking at 4 °C in distilled water (DW) (** *p* < 0.01) (Figure 1C). These results confirm that 4HGF release from the nanoparticles in a stimulated physiological condition.

### 2.2. Effects of PGA-4HGF on Primary Dermal Papilla Cells Proliferation and HaCaT Cells

**Figure 2:**
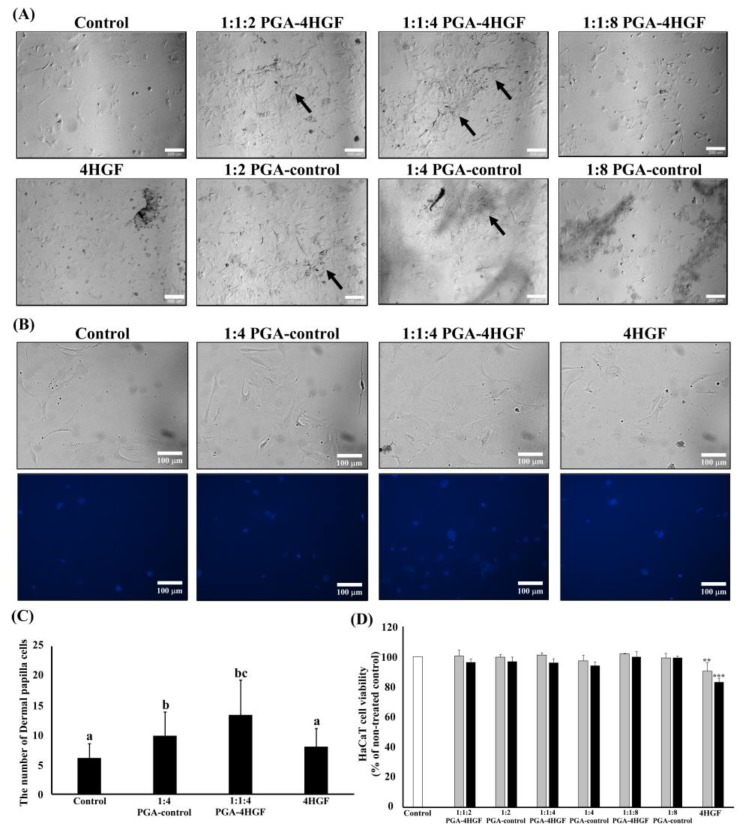
Effect of PGA-4HGF on primary dermal papilla cells and keratinocytes. (**A**) Primary dermal papilla cells (5 × 10^3^ cells/well) were treated with PGA-HGF for 96 h at 37 °C in Dulbecco’s modified Eagle’s medium (DMEM) media. The images of cell morphology and aggregation (black arrow) were taken after 96 h using a microscope (40× magnification, scale bars: 200 μm). (**B**) Primary dermal papilla cells (3 × 10^3^ cells/well) were co-incubated with various samples and Hoechst solution for 24 h. The images were taken after 24 h using a microscope (100× magnification, scar bars: 100 μm). (**C**) The number of DPCs were counted using Image J program. Data represent means ± deviation (SD). Data were analyzed with one-way ANOVA/Duncan’s *t*-test. (*p* < 0.05). Values with different alphabets in the same row are significantly different. (**D**) Cell proliferation effects of PGA-4HGF on keratinocytes (HaCaT) cells (5 × 10^3^ cells/well) in DMEM media for 24 h (grey bar: 1% of 4HGF, black bar: 2% of 4HGF). Each value represents the mean ± SD of three independent experiments. Data were analyzed with one-way ANOVA/Dunnett’s *t*-test (** *p* < 0.01, *** *p* < 0.001 vs. the non-treated control).

To elucidate whether PGA-4HGF induces the proliferation of primary dermal papilla cells (DPCs), we used different PGA-4HGF combination ratios (4HGF:PGA:chitosan = 1:1:2, 1:1:4, or 1:1:8) to treat primary DPCs. The proliferation and aggregation of primary DPCs was enhanced by 1:1:4 PGA-4HGF compared to 4HGF, non-treated control, and 1:4 PGA-control (Figure 2A, black arrow). To check whether PGA-4HGF had the ability to stimulate the stem cell like properties of DPCs, we observed cell morphology and aggregation. It is reported that DPCs have stem cell characteristics and are associated with the ability to induce hair-growth [26,27,28]. The flatten-elongated morphological changes and aggregation of primary DPCs were more prominent in 1:1:4 PGA-4HGF, compared with 4HGF, non-treated control, and 1:4 PGA-control (Figure 2A). In addition, The number of DPCs was significantly increased in 1:1:4 PGA-4HGF, compared with 4HGF, non-treated control and 1:4 PGA-control (Figure 2B,C). All PGA-4HGF samples had no effect on the viability of keratinocytes (HaCaT cells) (Figure 2D). Therefore, 1:1:4 PGA-4HGF was used for the rest of the study.

### 2.3. Hair-Growth Effects of PGA-4HGF in Telogenic C57BL/6N Mice

**Figure 3:**
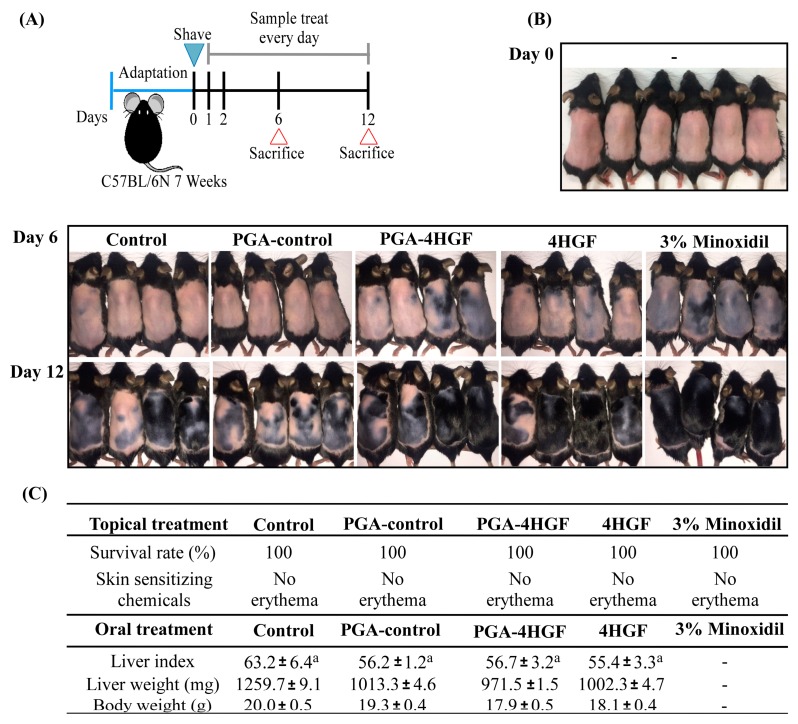
Hair-growth-promoting effects of PGA-4HGF in telogenic C57BL/6N mice. (**A**) The design of animal experiments using C57BL/6N telogenic murine models. At day 0 which a blue triangle is pointed, a 3 × 4 cm^2^ area of dorsal skin of all the mice were shaved. At day 6 and 12, which red triangles are pointed, the mice were sacrificed. (**B**) Dorsal skins were photographed on day 1 and 12. The images are representative pictures of the mice (*n* ≥ 6/group). (**C**) Survival rate of C57BL/6N telogenic murine models with PGA-4HGF topical treated during total animal experiments (*n* = 2). After PGA-4HGF treatment for 12 days in the C57BL6/N model, the analysis of the skin erythema status according to the standards announced by the Korea Food and Drug Administration. Body and liver weight of mice subjected to distilled water (control) gavage or PGA-4HGF Gavage. Data were analyzed with one-way ANOVA/Duncan’s *t*-test (*p* < 0.05). Each mean with a is not significantly different.

To determine the hair-growth promoting effect of PGA-4HGF in telogenic C57BL/6N mice, PGA-4HGF was topically applied on the shaved dorsal skin (Figure 3A). Black pigmentation was used as a biomarker for hair cycles, which changed from telogen to anagen phase [29]. By day 12, black areas were more prominent in the PGA-4HGF treated dorsal skins, compared with those in the non-treated control or PGA-control groups (Figure 3B). These results suggest that PGA-4HGF induced telogen to anagen conversion of the HFs.

To determine whether PGA-4HGF has adverse effects, we measured liver index after PGA-4HGF oral administration. The liver index of PGA-4HGF mice (56.7 ± 3.2) were similar to those of 4HGF mice (55.4 ± 3.3) (Figure 3C). No liver swelling and no body weight change was observed in all groups. Our data suggest that orally administered PGA-4HGF was not hepatotoxic.

**Figure 4:**
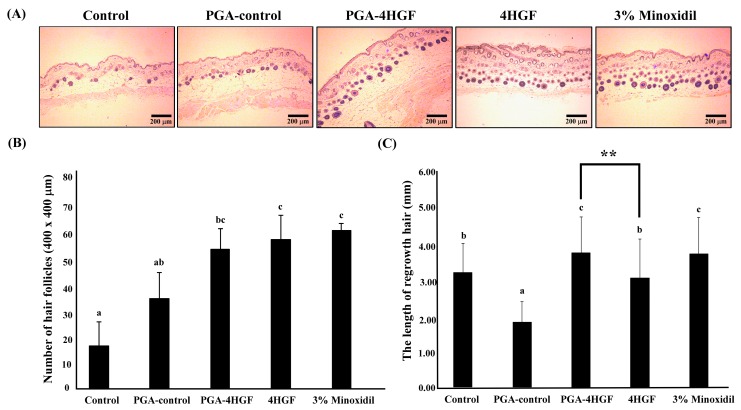
The effect of PGA-4HGF on hair follicles and hair length in telogenic C57BL/6N mice. (**A**) Transverse sections of the dorsal skins (12 days) stained with hematoxylin and eosin (H&E). Representative photomicrograph of the hair follicles in dorsal tissue treated with distilled water (DW), PGA, PGA-4HGF, 4HGF, or 3% minoxidil (200× magnification, scale bars: 200 μm). (**B**) The number of hair follicles of dorsal skins (400 × 400 μm^2^) and (**C**) the length of hair at 12 days after treatment with DW, PGA-control, PGA-4HGF, 4HGF, or 3% minoxidil. Data represent means ± deviation (SD) (*n* > 30 hairs). Data were analyzed with one-way ANOVA/Duncan’s *t*-test (*p* < 0.05). Values with a, b, c in the same row are significantly different. Data was analyzed with independent *t*-test (** *p* < 0.01).

The number of HFs per unit area (400 × 400 μm^2^) increased in PGA-4HGF-treated group (61.3 ± 11.36), compared with the number in the non-treated control (18.5 ± 3.15) and PGA-control (39.3 ± 10.67) groups (Figure 4A,B). The number of HFs in PGA-4HGF was not significantly different from that in 4HGF. By day 12, the regrown hair lengths of the PGA-4HGF treated group were 1.2-fold longer, compared with that of 4HGF group (Figure 4C). Our results indicate that PGA-4HGF induced hair-growth compared with the non-treated control and PGA-control groups and 4HGF groups.

### 2.4. Anagen Phase Induction in PGA-4HGF Treated C57BL/6N Mice

**Figure 5:**
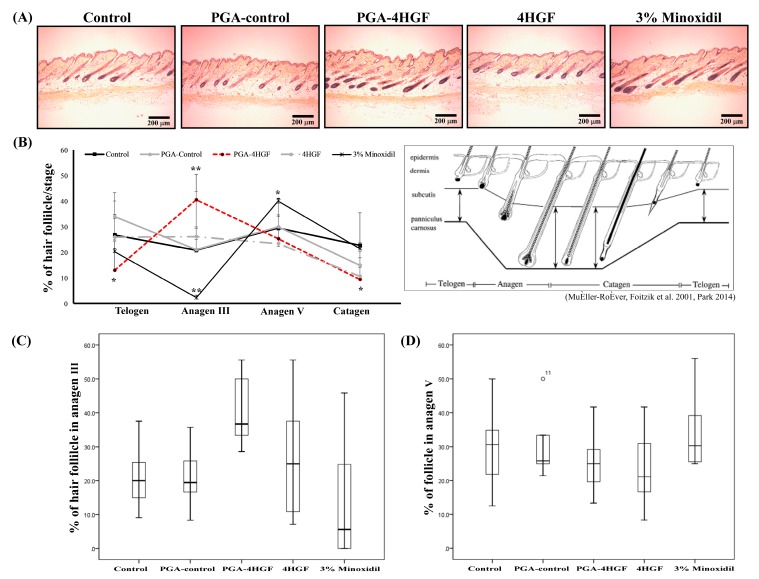
Early induction of the anagen phase by PGA- 4HGF treated in telogenic C57BL/6N mice. (**A**) The hair follicles in the longitudinal sections of the dorsal area were stained with H&E. Representative photomicrograph of the hair follicles in dorsal tissue treated with DW, PGA-control, PGA-4HGF, 4HGF, or 3% minoxidil for 12 days (200× magnification, scale bars: 200 μm). (**B**) Quantitative histomorphometric analysis. The percentage of the hair follicles in each hair cycle stage at 12 days was calculated (%: (number of hair follicles in each hair cycle/number of total hair follicles) × 100). Values data represent means ± standard error (SE). Data were analyzed with one-way ANOVA/Dunnett’s *t*-test (* *p* < 0.05, ** *p* < 0.01 vs the control group). Box-plot shows the % of hair follicle in anagen III (**C**) and the % of hair follicle in anagen V (**D**). The full line represents median values. Also shown are 10, 25, 75 and 90% percentiles of the variables.

The development of mouse HFs has been considered an indicator for the conversion of the HFs from telogen to anagen phase. To test whether PGA-4HGF induced anagen phase in hair cycle, the histological changes were analyzed after hematoxylin and eosin (H&E) staining of the tissues [5]. The histological data showed that the size and the number of the hair bulbs increased in the PGA-4HGF-treated group, compared with those in the 4HGF group (Figure 5A). At day 12, 40.5 ± 10.0% and 25.3 ± 9.2% of the HFs in PGA-4HGF-treated group had significantly progressed to the anagen III and V phases, while 26.2 ± 17.5% and 23.4 ± 10.6% of the HFs in the 4HGF group were in anagen III and V (*p* < 0.05) (Figure 5B). More HFs in the anagen phase were observed in PGA-4HGF groups (65.8 ± 19.2%) than in 4HGF groups (49.6 ± 28.1%). While the entire telogen phase HFs reside in the dermis, the anagen phase is related to the development and increase in the size and number of HFs residing in the deep subcutis (Figure 5A) [30].

### 2.5. Effects of PGA-4HGF on Wnt/β-Catenin Signaling and Its Related Cell Cycle Molecules

**Figure 6:**
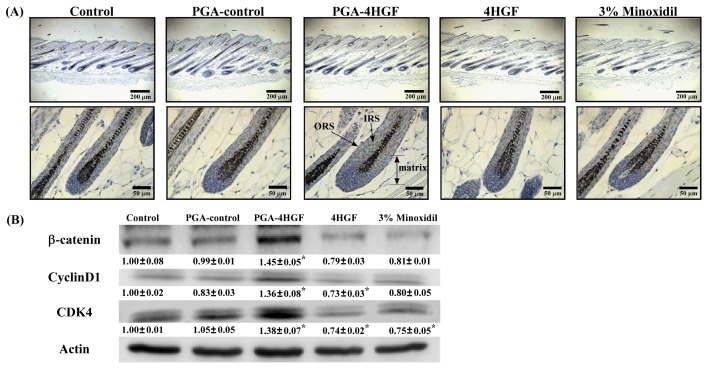
Induction of β-catenin and cell cycle-associated protein expression by PGA-4HGF in telogenic C57BL/6N mice. (**A**) Representative photomicrograph of the hair follicles in skin tissue treated with PGA-control, PGA-4HGF, or 4HGF, on day 12 (200 and 400× magnification, scale bars: 200 and 50 μm, respectively). (**B**) β-catenin, cyclinD1, and CDK4 protein expression levels in mice dorsal skin, detected using western blotting. Values data represent means ± standard error (SE). Data were analyzed with one-way ANOVA/Dunnett’s *t*-test (* *p* < 0.05 vs. the control group).

Hair-growth is mainly regulated by Wnt/β-catenin signaling that is involved in hair cycle and hair formation [31,32]. To evaluate whether PGA-4HGF affects the β-catenin signaling pathway, we verified the expression of Wnt/β-catenin signaling molecules, using IHC and western blotting. Our results revealed that β-catenin stained intensity in the PGA-4HGF-treated group was stronger than that in the 4HGF-treated group in the epidermis, outer root sheath (ORS), and hair matrix (Figure 6A). The distribution of β-catenin in the control group was primarily confined to the epidermis (Figure 6A). We observed that β-catenin protein expression was higher in the PGA-4HGF-treated group (1.45 ± 0.05), compared with the 4HGF group (0.79 ± 0.03) (Figure 6B). β-catenin plays an important role in DPCs proliferation and is also a transcription factor for the cell cycle-related proteins (cyclinD1/CDK4 complexes) [33,34]. CyclinD1 initiates progression from the G_0_ to G_1_ phase, which is activated by CDK4/6-dependent phosphorylation [35]. CyclinD1 and CDK4 protein expression levels were increased in the PGA-4HGF-treated group (1.36 ± 0.08 and 1.38 ± 0.07, respectively), compared with the 4HGF (0.73 ± 0.03 and 0.74 ± 0.02, respectively) group (Figure 6B). These results suggest that PGA-4HGF promotes hair-growth by upregulating β-catenin and inducing the G_1_ phase in the cell cycle, which affects the transition from the telogen to anagen phase.

### 2.6. Identification of Keratin Proteins in the Dorsal Skins of PGA-4HGF-Treated Mice Using Two-Dimensional Electrophoresis (2-DE) and Peptide Mass Fingerprinting (PMF)

**Figure 7:**
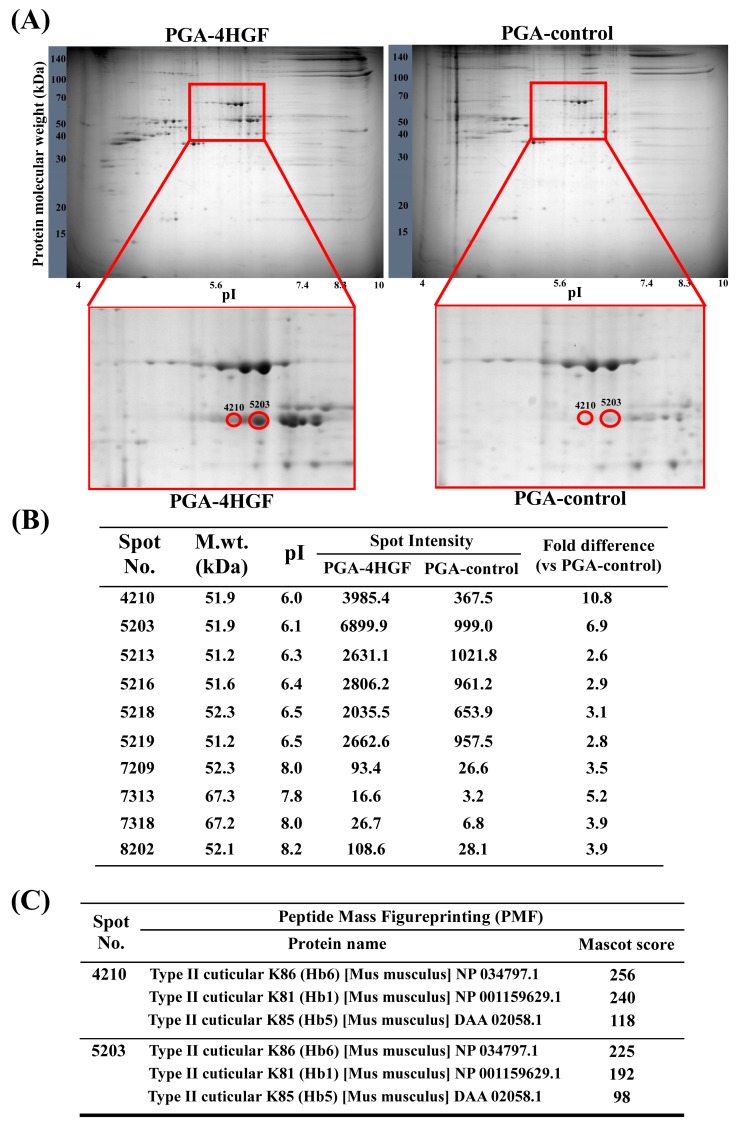
Characteristics of PGA-4HGF treated hair keratin proteins. (**A**) Two-dimensional electrophoresis (2-DE) separation of protein extracted from the dorsal tissues treated PGA-4HGF or PGA-control for 12 days using isoelectric focusing (IEF) strips and SDS-PAGE. (**B**) The protein spots detected from 2D gels and analyzed by the PDQuest (version 7.0, BioRad) software. The isoelectric point (pI) separation was done from pH 4 to 8. The molecular weight (M.wt) separation was done from 50–70 kDa. (**C**) The selected spots were analyzed using peptide mass fingerprinting (PMF).

To investigate for differences in the dorsal skin and HF keratin proteins between PGA-4HGF-treated and PGA-control mice, the hair proteins of murine dorsal skin were analyzed using 2-DE gel [36]. Only spots with a two-fold increase in intensity (compared to PGA-control group) were selected using the PDQest software (Figure 7A). The spot intensities of spots 4210 and 5203 were increased by 10.8 and 6.9-fold, respectively, in PGA-4HGF-treated mice, compared with the PGA-control group (Figure 7B). The selected spots (No. 4210 and No. 5203) were identified as type II keratin K81, K85, and K86 by peptide mass fingerprinting (PMF) [37]. The individual proteins are listed in Figure 7C. This analysis revealed that PGA-4HGF incresed the production of hair keratins such as type II keratin K81, K85, and K86 in the spot compared with the PGA-control. These result suggested that the type II cuticular (K81, K85, and K86) were 10.8 and 6.9-fold higher in the PGA-4HGF-treated group, compared with the PGA-control.

## 3. Discussion

Research on novel hair promoting agents is focused on effective natural products as well as carriers to improve and prolong hair-growth by enhancing penetration of effective components into hair follicles (HFs). Nanoparticles are known to efficiently transport drugs into skin areas with HFs [38,39,40]. PGA/chitosan nanoparticles are capable of entrapping biomolecules or agents in internal structures [22,24] and have a small size for enhancing drug delivery to HFs. The space between the hair follicle (20–50 μm) and shaft (16–42 μm) is approximately 200–400 nm, into which nanoparticles can conveniently fit [41,42]. We postulated that PGA-4HGF nanoparticles can get to the hair bulge in the stratum basal of the skin, avoiding the stratum corneum barrier because its size is ~400 nm (Figure 1A,B) [17,41,42]. PGA-4HGF nanoparticles in the hair bulge can improve blood circulation and in turn promote hair-growth [42]. To generate human HFs, DPCs should interact with themselves or other cell types (e.g., keratinocytes, dermal sheath cells, and follicular epithelial cells) [43]. We recently demonstrated that *C. militaris* grown on germinated soybeans (a 4HGF constituent) contains polyphenolic and flavonoid compounds which contribute to improve dermal blood circulation [44,45]. *P. multiflorum* and *Thuja orientalis* have been shown to increase β-catenin protein expression, involved in inducing telogen to anagen phase transition in the hair cycle [7,46]. In addition, PGA-4HGF nanoparticles can continuously release 4HGF for 6 h (Figure 1C), suggesting that PGA-4HGF is effective in prolonging 4HGF duration [15,22,23]. Therefore, PGA-4HGF nanoparticles, which are smaller in size, compared to HF pores, are increased in HFs compared to solely PGA or 4HGF, because 4HGF coupled to PGA can conveniently fit in the bulge or hair bulbs by PGA encapsulation.

DPCs play a key role in generating hair bulbs and regulating hair-growth [5,47]. They are known to aggregate reactions that induce HF formation, and determine the hair bulb and shaft due to stem cell-like plasticity [48,49,50,51,52]. Therefore, the proliferation and stemness of DPCs can increase hair-growth. As shown in Figure 2, 1:1:4 PGA-4HGF increased primary DPC proliferation compared with the other groups. PGA-4HGF also alters the shape of DPCs from spindle-shaped cells to flat multipolar and elongate-shaped cells and promotes DPCs aggregation, which are characteristics of active DPCs that can increase HF formation and prolong the anagen phase (Figure 2) [28]. Morphological changes and aggregation behaviors of PGA-4HGF-treated DPCs may be associated with the stem cell features in DPCs for hair formation [26,28]. Next, we also investigated whether 1:1:4 PGA-4HGF dose could promote hair-growth in an in vivo model.

The dorsal skin color of telogenic C57BL/6N mice is pink, but changes to dark black pigments during the anagenic phase [50]. Individual HFs can be divided into the specific hair cycle stages such as relative rest (telogen), active growth (anagen III–V), and involution, driven by controlled apoptosis (catagen), phases [53,54]. The HFs in the anagen phase have enlarged hair bulbs and large amounts of melanin around the upper half of the DPCs [53]. Melanin pigmentation is defined by melanogenesis, which involves transport of melanin granules to epidermal and hair follicle keratinocytes surrounding DPCs [55,56,57]. Melanin synthesis is related to hair-growth and hair shaft formation in the early anagen phase [50,58,59]. We observed more prominent black skin in the PGA-4HGF-treated groups, compared with the non-treated control and PGA-control groups (Figure 3B), suggesting that PGA-4HGF treatment can improve active growth (anagen phase).

The growing use of nanoparticles demands cautious evaluation of unexpected toxicities due to their physical and chemical characteristics [60]. It is reported that they can cut through the small intestines and spread extensively throughout the body [61]. We investigated if PGA-4HGF caused unwanted hepatotoxicity by observing liver images and calculating the liver index of each mouse after oral administration of PGA-4HGF. The average liver index of the PGA-4HGF-treated group (56.7 ± 3.2) was similar to that of the 4HGF-only group (55.4 ± 3.3) (Figure 3C). The mouse survival rate after oral or topical administration was 100% (Figure 3C). This is confirmation of no PGA-4HGF-associated abnormalities, as no liver damage and toxicity were observed.

Furthermore, it is reported that, as the cycle of HFs progresses from dormant to growth, the dorsal skin color turns to black and becomes more intense [53]. H&E staining results confirmed that the epidermis of mice treated with PGA-4HGF were darker and thicker, compared with the 4HGF group (Figure 4A). The PGA-4HGF-treated group produced a better effect on the length of regrowth hair (3.89 ± 1.04 mm), compared with the PGA-control group (1.89 ± 0.58 mm) (Figure 4B,C). In the telogen phase, HFs reside in the dermis and do not extend to the subcutis. Conversely, in the anagen phase, HFs reside in the deep subcutis and move closest into the panniculus carnosus in the late anagen phase. Based on previous research, we analyzed the HF cycle of each group and observed that a higher number of HFs in the PGF-4HGF-treated group were in anagen III and least in the telogen phase [53]. As a result, the PGA-4HGF-treated group converted approximately 15.3% of telogen follicles to anagen follicles, suggesting that it induced the anagen phase (Figure 5).

Hair is composed of type I and type II keratins, a family of fibrous structural proteins, containing 14–18% cysteine [62,63]. The existence of disulfide bonds in the keratin fiber complex determines the conservation of the strength, flexibility, and shape of HFs [63]. HFs in anagen phase consist of the cylindrical cell layers and the germinal matrix, which divide cells on the bulb [64]. Keratinocytes rapidly grow in the hair matrix zone surrounding the DPCs, which is stimulated by the β-catenin signaling pathway for the induction of hair-growth [65,66]. Interaction between keratinocytes and DPCs induce the expression of numerous genes encoding keratin intermediate filaments, such as type II keratin proteins [64,67,68]. Some reports showed that type II keratin genes are activated sequentially in DPCs [69]. According to previous studies, type II keratin K85, which constitutes the medulla, pre-cortex cuticle, and matrix, is expressed on the germinative compartment from the lower-most hair cuticle [67,70,71]. Type II keratin proteins K81 and K86 constitute the mid-cortex and upper medulla and are expressed in the medulla [62,67,72]. When keratin protein expression occurs improperly, this can cause hair diseases such as monilethrix, which is characterized by HF collapse and deformation, [62,70,73], ectodermal dysplasia, hypotrichosis, nail dystrophy, and hair scalp fragility [67,70,71]. In order to compare the expression of the hair-related proteins in mice treated with PGA-4HGF or PGA-control, 2-DE analyses using SDS-PAGE and PMF were performed. We observed that PGA-4HGF produced 10.8 and 6.9-fold more type II keratin proteins compared with the PGA-control group (Figure 7), suggesting that PGA-4HGF assisted in the formation of more durable HFs.

Previous studies have demonstrated that β-catenin induction in DPCs causes both hair-growth and regeneration [66]. β-catenin increases the proteins involved in stem cell functions [74]. Our previous data indicate that PGA-4HGF might increase β-catenin protein expression because it induces stem cell-like morphology in DPCs. β-catenin is also a transcription factor for cyclinD1 and CDK4 [3,34]. Most HF cells from bald patients are in the G_0_ phase [75]. CyclinD1 is known to induce G_1_/S phase transition [76,77,78,79]. Therefore, β-catenin is required for hair-growth, because it can induce the transition from the G_0_/G_1_ to the S phase [80]. We observed that PGA-4HGF increased the levels of β-catenin, cyclinD1, and CDK4 protein expression in the skin with hair (Figure 6). This data revealed that the down-stream targets of Wnt/β-catenin, cyclinD1, and CDK4 were upregulated in the PGA-4HGF-treated group. Herein, we observed that PGA-4HGF treatment increased the level of activated β-catenin (Figure 6), suggesting that the improvement of cell cycle progression in PGA-4HGF-treated cells can be attributed to the enhancement of the β-catenin pathway, since cyclinD1 and CDK4 are transcriptional targets of β-catenin that control cell cycle progression and induce cell proliferation.

In conclusion, we evaluated the hair promoting activity of PGA-4HGF in vitro and in vivo. Our results showed that PGA-4HGF induced DPCs proliferation, aggregation, and stem-cell like morphological changes, which may efficiently transport 4HGF into HFs. Overall, PGA-4HGF activates the β-catenin signaling pathway, leading to G_1_/S transition by increasing cyclinD1 and CDK4 protein levels, resulting in the increase of type II keratin proteins and melanin pigments, which promote durable hair formation. Therefore, we propose that the use of PGA nanocapsules for delivering 4HGF, may represent a promising therapy for treating hair-growth disorders.

## 4. Materials and Methods

### 4.1. Preparation and Characterization of the 4HGF Loaded Nanoparticles (PGA-4HGF)

4HGF (The mixture of *P. linteus* grown on germinated brown rice, *C. militaris* grown on germinated soybeans, *P. multiflorum*, *F. carica*, *C. nucifera* oil, etc.) was kindly provided by CARI Co. Ltd. To entrap 4HGF into PGA hydrogel, the 4HGF mixture was filtered through filter paper Whatman’s No. 1 and then centrifuged to obtain the supernatant and sedimented at 1630× *g* for 10 min (Union 32R, Hanil Science Industrial Co., Incheon, Korea). PGA-4HGF hydrogel nanoparticles were prepared through simple ionic gelation under shaking condition at room temperature. In brief, hair growth solution was thoroughly mixed with poly γ-PGA solution (1 mg/mL), subsequently dropped into aqueous chitosan solution (1 mg/mL) at room temperature with stirring for 1 h. Initially, hair growth solution with poly γ-PGA solution were added to several ratios of chitosan (1:1:2, 1:1:4, and 1:1:8). After the hydrogel nanoparticles were synthesized, they were separated via centrifugation at 10,000× *g*, then dispersed in distilled water at 4 °C until use. The size and morphology of the prepared nanoparticles were analyzed by transmission electron microscopy (TEM) images on a Jeol EM-2010 microscope (Jeol Co., Peabody, MA, USA) and dynamic light scattering using a Zetasizer Nano-ZS (Malvern Co., Malvern, UK).

### 4.2. HGF Release from the Nanoparticles (PGA-4HGF)

The released amount of 4HGF from the hydrogel nanoparticles was evaluated by measuring the absorbance at 400 nm of the supernatant solution, which corresponded to the 4HGF. The shaking incubator was used to evaluate the released amount of 4HGF from hydrogel nanoparticles. Firstly, 400 µL DW or PBS (10 mM, pH 7.4) was added to the tube which contained 200 µL of nanoparticle mixture. The mixture was incubated for 6 h in the shaking incubator (37 °C, 200 rpm). Then, the mixture was centrifuged, and the supernatant was used to measure absorbance at 400 nm to determine the amount of 4HGF released from the hydrogel nanoparticles, based on the standard calibration plot.

### 4.3. Cell Culture and Proliferation of Primary DPCs Using Real Time Microscopy

Primary DPCs were isolated from the follicle bulbs of C57BL/6N mice whiskers as previously described [81,82]. The isolated DPCs were cultured in Dulbecco’s modified Eagle’s medium (DMEM; Invitrogen Co., Carlsbad, CA, USA) with 100 units/mL each of penicillin A and streptomycin (Gibco BRL, Grand Island, NY, USA), and 10% heat-inactivated fetal bovine serum (FBS; Gibco BRL Grand Island, NY, USA). Cells were grown at 37 °C in fully humidified 5% CO_2_ (Forma 3111, Thermo Fisher Scientific, Waltham, MA, USA).

The primary DPCs (5 × 10^3^ or 3 × 10^3^ cells/well) were seeded onto 24-well plates. PGA-4HGF (2% (*v*/*v*)) (4HGF:PGA:chitosan = 1:1:2, 1:1:4, or 1:1:8), PGA-control (PGA:chitosan = 1:2, 1:4, or 1:8), and 4HGF were treated to DPCs, and images of DPC proliferation (40× magnification) were taken after 96 h using a CCD camera (Point Grey Research Inc., Richmond, BC, Canada) and analyzed using the MetaMorph software (Universal Imaging, West Chester, PA, USA).

To quantify the number of DPCs, 1:1:4 PGA-4HGF (2% (*v*/*v*)), 1:4 PGA-control, and 4HGF were treated to DPCs with Hoechst 33258 solution (Thermo Fisher Scientific, Waltham, MA, USA). The image of stained DPCs (100× magnification) was taken after 24 h (Nikon Eclipse Ti, Nikon Instruments Inc., Kobe, Japan) and then was analyzed using the Image J software (Wright Cell Imaging Facility, version, city, if any state, country).

### 4.4. Cell Proliferation of Human HaCaT Cells

HaCaT cells (5 × 10^3^ cells/well), human normal keratinocytes, were cultured in 96-well plates containing DMEM (100 units/mL), penicillin A and streptomycin, and 10% heat-inactivated FBS. These cells were also grown at 37 °C in fully humidified 5% CO_2_. We used the PGA-4HGF with different capture solution ratios (4HGF:PGA:chitosan = 1:1:2, 1:1:4, or 1:1:8). PGA-4HGF (1% and 2% (*v*/*v*)) was added to HaCaT cells. Cell proliferation was measured using the cell count kit-8 assay (Ez-Cytox kit; Daeil Lab service, Seoul, Republic of Korea). Ez-Cytox solution (10 μL) was added to each well and the cells were incubated at 37 °C for 2 h. The absorbance was measured at 450 nm using a microplate reader (Epoch; Biotek Instruments, Inc.).

### 4.5. Anagen Phase Induction in C57BL/6N Mice

Female 7-week-old C57BL/6N telogenic mice were purchased (Orient Bio, Eumsung, Republic of Korea) and maintained under specific pathogen free (SPF) conditions with 12 h light/darkness cycles. Six mice were randomly divided into 1 of 5 groups and allowed to acclimatize to laboratory conditions for 7 days. Mice were fed with the standard diet and allowed free access to drinking water. A 3 × 4 cm^2^ area of dorsal skin of all the mice were shaved using attenuated hair removal cream (BIKIRO cream (Thioglycolic Acid 80%); Tai Guk Pharm. Co. Ltd., Gyeonggi-do, Republic of Korea). Samples were applied daily on the shaved dorsal skin hair. As initially indicated, samples received: 200 μL of control (distilled water, DW), PGA-control (PGA:chitosan = 1:4), PGA-4HGF (4HGF:PGA:chitosan = 1:1:4), 4HGF, and 3% minoxidil (Dongsung, Seoul, Republic of Korea). Superficial properties of hair growth were measured and photographed on day 12 of PGA-4HGF treatments (Figure 3A). All researchers on the animal studies were complied with the standards for the care and use of experimental animals. The animal study was performed in accordance with the Institutional Animal Care and Use Committee (IACUC) guidelines at Gachon University (approval number: GIACUC-R2016022, approval Date: 31 October 2016).

### 4.6. Toxicity Test of PGA-4HGF

γ-PGA/chitosan and PGA-4HGF nanoparticles were orally administered to the mice at a dose of 2 mL/kg. The control mice received 2 mL/kg DW. A day after oral administrations of treatment, the mice were sacrificed, and their livers recovered and weighed. Liver index was calculated as the weight of each liver divided by the total body weight (g). All researchers on the animal studies were complied with the standards for the care and use of experimental animals. The animal study was performed in accordance with the Institutional Animal Care and Use Committee (IACUC) guidelines at Gachon University (approval number: GIACUC-R2016021, approval Date: 31 October 2016).

### 4.7. Hair Follicle Counting and Hair Length Determination

Digital photomicrographs were taken from representative areas of the dorsal skin tissue slides at a fixed 40x magnification. All images were chopped in a fixed area (400 × 400 μm^2^). The HFs in deep subcutis were manually counted (*n* > 30/mouse). The regrown hairs were plucked from the dorsal skin areas (100 × 300 mm^2^) and the hair length of each sample calculated (*n* > 30/mouse).

### 4.8. Histological Preparation and Hematoxylin-Eosin Staining

Dorsal skin tissues from each mouse were fixed in 10% formaldehyde for 24 h and embedded in paraffin blocks. They were cut transversely or longitudinally into 4 μm thick sections and mounted on glass slides. To observe for histological changes, the slides were stained with H&E staining solution. The slide images were taken under a Nikon Eclipse Ti microscope equipped with a color digital camera (Point Grey Research, Richmond, BC, Canada) and analyzed using MetaMorph software (Molecular devices, Sunnyvale, CA, USA).

### 4.9. Immunohistochemistry

As previously described [5], the dorsal skin was stained with anti-β-catenin (Cell Signaling, MA, USA) antibodies post topical PGA-4HGF treatment. To extinguish endogenous peroxidase activity, de-paraffinized parts were pre-treated with 0.3% H_2_O_2_ for 10 min. After washing with Tris-buffered saline containing Tween (TBS-T), the sections were incubated with 4% bovine serum albumin(BSA) with dextran for 30 min to prevent nonspecific binding of the secondary antibody, and incubated with anti-β-catenin (1:400 dilution) antibodies for 1 h. Slides were incubated with anti-rabbit biotin secondary antibody (Agilent, CA, USA) for 30 min. The slides were counter-stained with Mayer’s hematoxylin for 1 min, viewed under a Nikon Eclipse Ti microscope equipped with a color digital camera (Point Grey Research, Richmond, BC, Canada), and analyzed using the MetaMorph software (Molecular devices, Sunnyvale, CA, USA).

### 4.10. Western Blot Analysis

Western blotting was performed as previously described [45]. Briefly, the tissues were crushed in a tissue homogenizer after being lysed in radioimmunoprecipitation assay (RIPA) buffer (Cell Signaling, MA, USA). The proteins were then separated by centrifugation at 14,000× *g* for 10 min. Protein concentrations were determined using the Pierce bicinchoninic acid (BCA) protein assay kit (Thermo Fisher Scientific, Waltham, MA, USA). Equal protein amounts were separated using 10% sodium dodecyl sulfate polyacrylamide gel electrophoresis (SDS-PAGE). Proteins were transferred to nitrocellulose membranes (Bio-Rad Laboratories, Inc., Hercules, CA, USA) and blocked with 5% non-fat milk for 1 h at room temperature. They were then incubated overnight at 4 °C with Tris-buffered saline containing Tween (TBS-T, 20 mM Tris, 500 mM sodium chloride (pH 7.6), and 0.1% Tween 20), and 5% bovine serum albumin, anti-*β*-catenin (1:1000; Cell Signaling, MA, USA), anti-cyclin D1 (1:1000; Abcam, Cambridge, UK), and anti-CDK4 (1:1000; Abcam, Cambridge, UK). The membranes were washed 3× for 10 min with TBS-T and then incubated for 1 h with horseradish peroxidase (HRP)-linked anti-rabbit IgG (1:2000; Cell Signaling, MA, USA). The blots were detected using an enhanced chemiluminescence western blotting detection system with the Odyssey LCI Image software (LI-COR Biosciences, Lincoln, NE, USA). The blots shown are representative of at least 3 repeats.

### 4.11. Sample Preparation for Keratin Protein Analysis

Dorsal skin tissues were washed twice with ice-cold PBS, placed in a tissue lysis solution (7 M urea, 2 M Thiourea containing 4% (*w*/*v*) 3-[(3-cholamidopropy) dimethyammonio]-1-propanesulfonate (CHAPS), 1% (*w*/*v*) dithiothreitol (DTT), 2% (*v*/*v*) pharmalyte and 1 mM benzamidine) and immediately homogenized with a motor-driven homogenizer (PowerGen125, Fisher Scientific). Proteins were extracted after vortexing for 1 h at room temperature and centrifugation at 15,000× *g* for 1 h at 15 °C. The soluble fraction was used for two-dimensional gel electrophoresis. Protein concentration was measured using the Bradford assay [83].

### 4.12. Protein Identification by Two-Dimensional Electrophoresis (2-DE) and Peptide Mass Fingerprinting (PMF)

IPG dry strips (4-10 NL IPG, 24 cm; Genomine, Republic of Korea) were equilibrated with a mixture of 7 M urea and 2 M thiourea (containing 2% CHAPS, 1% DTT, and 1% pharmalyte) for 12–16 h. Sample (200 μg) was then loaded in each well. Isoelectric focusing (IEF) was performed using a Multiphor II electrophoresis unit and EPS 3500 XL power supply at 20 °C. Equilibrated strips were run in the Hoefer DALT 2D system, following the manufacturer’s instructions (Amersham Biosciences, Uppsala, Sweden). Gels (2D) were stained using Colloidal Coomassie Brilliant Blue as described by Oakley et al. [84]. Quantitative analyses of digitized images were carried out using the PDQuest (version 7.0, BioRad, city, if any state, country) software, following the manufacturer’s instructions. The quantity of each spot was normalized by total valid spot intensity. Protein spots were only selected if they showed at least 2-fold increased protein expression values compared to the PGA-control.

For peptide mass fingerprinting, protein spots were excised, and digested with trypsin (Promega, Madison, WI), mixed with α-cyano-4-hydroxycinnamic acid in 50% acetonitrile/0.1% trifluoroacetic acid (TFA), and subjected to MALDI-TOF analysis (Microflex LRF 20; Bruker Daltonics, Billerica, MA) as described by Fernandez et al. [37]. The search program MASCOT (Matrixscience, available at www.matrixscience.com), was used for protein identification. The following parameters were used for the database search: trypsin as the cleaving enzyme, a maximum of one missed cleavage, iodoacetamide as a complete modification, oxidation as a partial modification, monoisotopic masses, and a mass tolerance of ± 0.1 Da. The PMF acceptance criteria were probability scoring. Keratin proteins were detected as described by Schweizer [71,85].

### 4.13. Statistical Analysis

Data were obtained from at least 3 independent experiments and presented as mean ± standard deviation (SD). Statistical analyses were performed using the student *t*-test, paired *t*-test and one-way ANOVA with Dunnett’s or Duncan’s post-hoc tests. Data were analyzed using the SPSS v12 software (Chicago, USA).
